# The Influence of Nanographite Addition on the Compaction Process and Properties of AISI 316L Sintered Stainless Steel

**DOI:** 10.3390/ma15103629

**Published:** 2022-05-19

**Authors:** Barbara Kozub, Marimuthu Uthayakumar, Jan Kazior

**Affiliations:** 1 Department of Materials Engineering, Faculty of Materials Engineering and Physics, Cracow University of Technology, Al. Jana Pawła II 37, 31-864 Cracow, Poland; kazior@mech.pk.edu.pl; 2 Faculty of Mechanical Engineering, Kalasalingam Academy of Research and Education, Krishnankoil 626126, India; uthaykumar@gmail.com

**Keywords:** AISI 316L, sintering, nanographite, BET specific surface area, Kenolube, stearic acid

## Abstract

This paper presents the effect of graphite addition on the pressing process and selected mechanical properties of AISI 316L austenitic stainless steel. The graphite powders used in this study differed in the value of the specific surface area of the particles, which were 15 (micropowder), 350, and 400 m^2^/g (nanopowder). Mixtures with the addition of lubricants—stearic acid and Kenolube—were also created, for comparison purposes. The scope of the tests included compressibility of blends, measurements of the ejection force while removing the compacts from the die, micro-structural studies, a static tensile test, a three-point bending test, a Kc impact test, Rockwell hardness, and Vickers microhardness measurements. The study demonstrated that the addition of graphite nanopowder to the studied steel acts as a lubricant, providing a significant improvement in lubricity during the pressing process. Moreover, the addition of nanographite allowed for a significant increase in the mechanical properties studied in this work; it was observed that, for the sinters made of mixtures with a higher graphite content and with a large specific surface area of its particles, better values for the tested properties were obtained.

## 1. Introduction

Powder metallurgy technology (PM) belongs to complex manufacturing processes. Each stage, including selecting powders and lubricants, mixing, pressing, sintering, possible re-pressing, and re-sintering, must be precise to obtain the appropriate chemical composition and structure—and consequently, the appropriate technological properties of the sinter [1,2]. Molinari et al. [1,3], in their works, state that the most sensitive to interference and, at the same time, the most challenging stage of PM is the sintering process, which is connected with the necessity of simultaneous selection of many technological parameters directly influencing the sintering process, i.e., heating and cooling rates, selection of the lubricants and their removal temperatures, temperature and time of isothermal sintering, and the applied sintering atmosphere. Analyzing the research results presented in numerous scientific papers [1,2,3,4,5,6,7,8], it can be concluded that, among the above-mentioned technological parameters, selecting an appropriate sintering atmosphere has a significant influence on many chemical and physical aspects of the sintering process. Foremost, the used atmosphere must enable the complete removal of the lubricants and the reduction of the oxide layer from the surface of the sintered material particles, which is a necessary condition for the sintering process to begin. In addition, an appropriate sintering atmosphere for stainless steel allows for avoiding the formation of chromium compounds that affect the reduction of corrosion resistance [1].

In the case of sintering compacts made of high chromium metal alloy powders, another significant factor is the issue of the purity of the sintering atmosphere. According to Danninger [9], impurities present in the chosen atmosphere, such as O_2_, H_2_O, etc., react violently with the sintered material, causing the formation of an oxide layer on the powder particle surfaces. At the same time, Larsen [8] and Lindqvist [10], in their studies, indicate that, to prevent oxidation, the partial pressure of oxygen in the applied sintering atmosphere must not exceed 10^−17^ Pa.

It should also be remembered that the correct choice of powder chemistry and sintering atmosphere for sintered stainless steel is significant. Terrissi [4] has studied and described the negative effect of high carbon content resulting in the formation of carbide inclusions, which significantly reduces the corrosion resistance of steel. The author also highlighted the important role of nitrogen and oxygen in shaping the properties of sintered stainless steel. At the same time, too high of a nitrogen content, with a poorly chosen cooling rate, can result in the formation of nitrides, which adversely affect the corrosion resistance of stainless steel [4]. Probably the least studied issue is the influence of oxygen on the corrosion resistance behavior of sintered stainless steel. Numerous researchers, including Larsen and Thorsen [8,11], Pao and Klar [12], Terrisse et al. [4], Tunberg et al. [5], and Beste [13], presented research in their works indicating strong relations between oxygen content and the mechanical properties and corrosion resistance of sinters. These studies suggest that an increase in oxygen content is generally associated with an increase in yield strength; unfortunately, at the same time this causes a deterioration in corrosion resistance and a decrease in the ductility and strength of sintered stainless steels. Furthermore, the natural oxygen content of powders is often increased due to continuous storage in, or temporary contact with, air. There is adsorbed oxygen on the surface layer of powder particles in the form of O_2_, hydroxides, water, and/or metal oxides, which must be eliminated during the sintering process [14,15,16].

One of the indispensable aspects of powder metallurgy technology is the issue of lubrication. Although the volume proportion of lubricants in the powder mixture is small, they significantly affect the powder densification process. The friction created between the die walls and the molded part impedes pressure transfer, resulting in an apparent density gradient of the molded part [17,18,19]. Therefore, using lubricants can reduce the inhomogeneity of density distribution by promoting uniform pressure transfer during the pressing process. In addition, lubricants affect the matrix filling density and powder flowability and, consequently, the consolidation characteristics of the powder particles during the compaction as well as the density and strength of the compacted materials [20,21]. At the same time, the reduction in friction between the compressed molded part and the die walls allows the compacts to be pushed out of the die more easily, which ultimately leads to reduced wear [19,22].

Conventional lubricants used for cold pressing include synthetic amide waxes, metal stearates, polyethylene waxes, and composite hybrids of stearates and waxes mixed with metal powders in the amount of 0.5 ÷ 1.5%, by weight [19,22,23,24,25]. Żółkowski and Czepelak, in their work [26], mention the boiling point (or decomposition temperature), degree of disintegration, moisture content, and size of the decomposition residue as the basic and decisive properties of lubricants. The simplest method of lubrication is to add a lubricant, in sufficient quantity, to the base powder at the stage of mix preparation. This method extends the life of expensive tools by reducing stresses between particles. Note that the introduction of a lubricant into the powder mixture can negatively affect the density of the molded part; the lubricant particles fill the empty spaces between the base powder particles, and thus hinder the pores from being supplied through the plastic deformation mechanism, as Simchi [27] and Rahman [28] demonstrated in their study. At the same time, the admixture of lubricants results in much less metal–metal bonding during compaction, which negatively affects the strength of the molded part. It is also important to note that, after pressing, the lubricant must be removed from the molded part during sintering [19,28]. Using a suitable sintering atmosphere and with a controlled heating rate, the removal of lubricants usually occurs in the temperature range of 400 °C to 600 °C. The mechanism of the lubricant removal process has been quite well described in the literature [22,23,29,30]. In their works, Barrow [22], Saha [23], Simchi [27], Mares [29], and Lindskog [30] distinguish between several stages of lubricant removal: melting, evaporation, and vapor diffusion through the pore network to the surfaces of the sintered part, as well as lubricant vapor removal by gas flow in the furnace. The same authors show that improperly carried out lubricant removal can lead to defects, such as blistering, soot deposition, microporosity, and carbon segregation. On the other hand, Simchi [19] and Rahman [28], in their publications, addressed the problem of lubricant removal from high-density moldings, which is more difficult because of the gas pressure of lubricant evaporation during the sintering process; this can create voids of significant size, which in turn can adversely affect densification during sintering. Therefore, to prevent the variable apparent density distribution of the molded part, as well as the problems associated with doping of the lubricant, the PM industry always tries to select an amount of slip agent that preserves the advantages of its use.

The behavior of graphite as a lubricant is reasonably well understood and described in the literature [29,31,32,33]. Graphite is considered to be an effective particulate-based lubricant. However, as investigated and described by Mares and Tamashausky [29], for a typical amount of graphite introduced into materials obtained by PM technology, which ranges from 0.7% to 0.9% by weight, the ratio of graphite flake area to die wall area is insufficient to produce a lubricating layer covering the die walls, which does not allow efficient pushing of the molded part out of the die. Nanographite, having a reduced number of graphene planes, provides improved lubricity when exposed to shear or other forces that would typically form a lubricating film. For example, Mares and Tamashausky report that a synthetic graphite powder with a particle size of 8 μm will have a BET (Brunauer–Emmett–Teller theory-based technique of the specific surface area measurement [34]) specific surface area of 11 m^2^/g.

In contrast, a nanographite powder with a reduced nominal particle size of 100 nm will have a much larger BET specific surface area of 350 m^2^/g (this is an “apparent” size because agglomerates of much finer primary particles are formed in the graphite powder). Such a significant difference in the size of the BET specific surface area is because, regardless of the abrasion method, the thickness of the remaining graphite plates decreases as smaller particles are formed. The number of particles per unit mass increases significantly with a resulting decrease in bulk density [29,31,32,33].

The influence of graphite nanopowder addition to the AISI 316L austenitic stainless steel on the pressing process and selected mechanical properties of sinters was investigated. The graphite powders used in this study differed in the value of the specific surface area of the particles, which were 15, 350, and 400 m^2^/g. Compounds with the addition of the lubricants stearic acid and Kenolube were also made, for comparison purposes. Tested samples were sintered in a vacuum technical atmosphere, which is an alternative to sintering the tested steel in an atmosphere showing reducing properties, e.g., one containing hydrogen, which is known to be dangerous and is not always applicable in industrial settings. The legitimacy of the studies, the results of which are presented in this paper, is confirmed by literature reports, which show that the micrographite addition to stainless steel, depending on its percentage in the mixture and selected parameters of the sintering process, does not always allow the reduction of oxides during sintering at a satisfactory level. Since nanographite powders have a highly developed BET specific surface area, it can be speculated that the reduction reaction of oxides will be intensified during the sintering process, which will contribute to the faster exposure of pure metallic surfaces, thus reducing the porosity in the sinter. This should have a measurable effect in improving the properties of the sintered material. At the same time, no existing studies address the practical aspect of introducing graphite nanopowder to austenitic stainless steel powder. It can be assumed that this procedure will have a positive effect on the density of the powder, and will reduce the forces pushing the molded parts out of the matrix; the nanographite, apart from playing the role of an activator (as shown in an earlier published paper [35]), will also play the role of a lubricant, creating a lubricating film between the powder particles and the walls of the matrix. This opens up the possibility of eliminating commercially available lubricants that, when introduced into stainless steels, often cause negative effects on the final properties of sintered products. From a practical point of view, this is an undoubted economic advantage, primarily due to the reduction of sintering time by eliminating the step of removing the lubricants.

Furthermore, it is essential to note that an improperly performed lubricant removal process can lead to defects, such as blistering, soot deposition, microporosity, and carbon segregation, in the sinter. On the other hand, large, individual gas-filled pores may form during the removal of slip agents from high-density moldings, due to the high pressure of the gaseous products of their thermal decomposition. In addition, incompletely removed lubricants may cause a decrease in the surface activity of the sintered steel particles, resulting in a reduction in the degree of sinter densification.

At the same time, it is worth noting that the scientific papers published so far present the results of research conducted for various technological conditions of sintering production, including different pressing pressures, shapes, and sizes of samples, different amounts of graphite powder added to the stainless steel, and different temperature and time parameters of the sintering process, which significantly hinders the comparative analysis of the published research results. Moreover, most authors emphasized the analysis of oxide layer reduction processes, exclusively, during the graphite-doped stainless steel sintering process. In selected works, one can find measurements of the degree of density and corrosion resistance of sinters. Only a few are devoted to studying these sintering methods’ effect on the sinters’ mechanical properties.

## 2. Materials and Methods

### 2.1. Materials

Powder mixtures with the AISI 316L austenitic stainless steel powder, produced by Höganäs AB, Sweden, as a base material were made for the study. The chemical composition of the AISI 316L powder used is presented in Table 1. The flowability and bulk density of the used AISI 316L powder are 29.1 s/50 g and 2.90 g/cm^3^, respectively. The following were used as modifying additives for the stainless steel:

- GS-TC307 nanographite powder, which has a specific surface area of 350 m^2^/g, from Graphitestore (Northbrook, IL, USA);
- GS-TC307 nanographite powder, which has a specific surface area of 400 m^2^/g, from Graphitestore (Northbrook, IL, USA);
- TIMREX F10 PM Special Graphite flake micrographite, which has a specific surface area of 15 m^2^/g, from TIMCAL (Bodio, Switzerland).

Graphite powders were introduced in amounts of 0.1, 0.2, and 0.3%, by weight.

For comparison purposes, blends of the investigated stainless steel powder with the addition of lubricants, introduced in the amount of 0.6% by weight, were made. The following lubricants were used to make the mixtures: Kenolube P11 (Zn-containing organic lubricant) and stearic acid. Table 2 presents the characteristic features of the lubricants used in the tests.

The procedure of preparing the materials for the tests included the preparation of powder mixtures, by introducing the assumed amounts of additives (0.1, 0.2, and 0.3%, by weight, of graphite powders, and 0.6%, by weight, of lubricants) into the AISI 316L austenitic stainless steel base powder. The prepared powder mixtures were mixed for 12 h using a Turbula T2F mass mixer (Glen Mills Inc., Clifton, NJ, USA).

The composition of the individual mixtures tested at work and the adopted designation are presented in Table 3.

### 2.2. Testing Methods

Three cylindrical specimens of Ø20 mm diameter were made for each prepared mixture for a constant mass of 10 g. The weights were pressed on one side on Otto HS WWe-100 V.1 hydraulic press (Otto HS, Reda, Poland) in the pressure range from 200 MPa to 800 MPa (step 100 MPa). Then, to prepare the density curve, the densities of the obtained moldings were determined. The density was measured using a geometric method (based on sample mass and dimensions measurements).

Measurement of forces of the ejection of compacts from the die for all tested compositions of mixtures was carried out on the hydraulic press EU 20, equipped with instrumentation for recording and processing measurement signals. To obtain uniform conditions for the measurements of forces of compacts ejection from the die, i.e., the same areas of cylindrical surfaces of the samples, five compacts of constant volume were made for each of the mixtures. The mixture weights were pressed unilaterally in the die, with the diameter of the filling hole equal to Ø20 mm at 600 MPa pressure. Five repetitions of measurements were carried out for all compositions of the mixtures.

The densities of the compacts and sinters were calculated based on their weight and dimensions (geometric method). The sinters’ apparent density and porosity, including closed and open porosity, were determined by the Archimedes method following the PN-EN ISO 2738:2001 standard [37].

**Figure 1:**
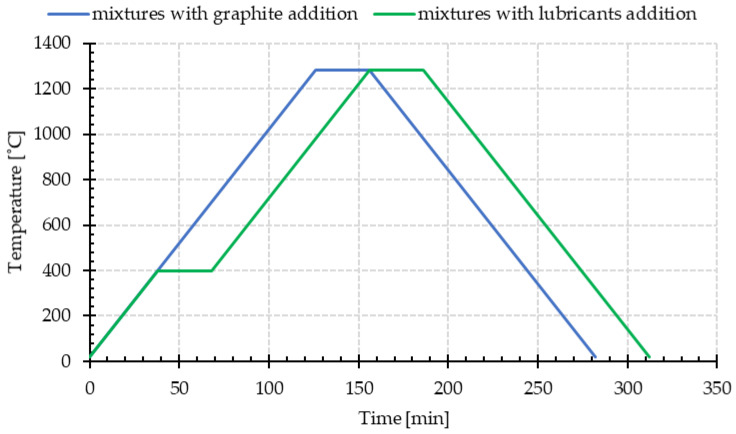
The temperature profiles of the sintering process for the tested mixtures.

The samples were sintered at the temperature of 1280 °C for 30 min in an atmosphere of technical vacuum (10^−2^ Pa), at 10 °C/min for heating and cooling. Samples made from base powder blends with stearic acid and Kenolube were additionally annealed at 400 °C for 30 min, due to the necessity of removing the sliding agents before isothermal sintering. Figure 1 shows the adopted temperature profiles of the sintering process for the tested mixtures.

The microstructure of the sinters was tested on metallographic samples prepared following the metallographic procedure. The metallographic specimens were heat-etched with Villel’s reagent, which consisted of 30 mL glycerin, 10 mL nitric acid HNO_3_, and 20 mL hydrochloric acid HCl. Microstructure studies of the sintered additive-modified stainless steels were carried out on specimens in the un-etched state, as well as after etching, using a Nikon Eclipse ME 600 optical microscope with digital image recording (Nikon Corporation, Minato, Tokyo, Japan).

The static tensile test was carried out on an MTS Insight 50 testing machine (MTS System Corp., Eden Prairie, MN, USA). The elongation of the specimens, with standardized dimensions (PN-EN ISO 2740:2010 [38]), was measured using an MTS resistance extensometer. The average strain rate during the test was 0.0001 1/s. The tensile strength (R_m_), conventional yield strength (R_0.2_), relative elongation (A_5_), and Young’s modulus (E) were determined for all the sinters investigated based on the static tensile test.

For impact testing, three series of 55 × 10 × 10 mm unnotched rectangular specimens were made following PN-EN ISO 5754:2018-02 [39]. Impact testing was performed on a 150J Toropol impact hammer (ToRoPoL, Warsaw, Poland).

Three series of 5 × 10 × 35 mm cuboid shapes were made to measure the bending strength of the tested sinters. Static three-point bending was carried out according to PN-EN ISO 3325:2000 [40] on an MTS Criterion 43 testing machine (MTS System Corp., Eden Prairie, MN, USA). The measurement base of the samples was 20.5 mm. The measurements were conducted at a preset speed of 0.5 mm/min until the cohesion of the specimens broke.

In addition, breakthroughs of impact specimens and breakthroughs of specimens after static tensile testing and three-point bending were observed. Images of the fractures were taken using a JOEL JSM5510LV scanning electron microscope (JOEL Ltd., Tokyo, Japan).

The sinter hardness, measured on the surface from the upper and lower punch, was tested using the B-scale Rockwell method (980.7 N main load), on an Innovatest CU-600MBDL hardness tester (INNOVATEST, Halesowen, UK), according to the PN-H-04938:1978 [41] standard. Meanwhile, microhardness measurements were performed using the Vickers method, according to the PN-EN ISO6507-1:2018-05 [42] standard, on an Innovatest 423A microhardness tester (INNOVATEST, Halesowen, UK) with a loading force of 0.05 N. The hardness and microhardness values reported in this paper were arithmetic averages obtained from ten measurements.

## 3. Results and Discussion

### 3.1. Compressibility

**Figure 2:**
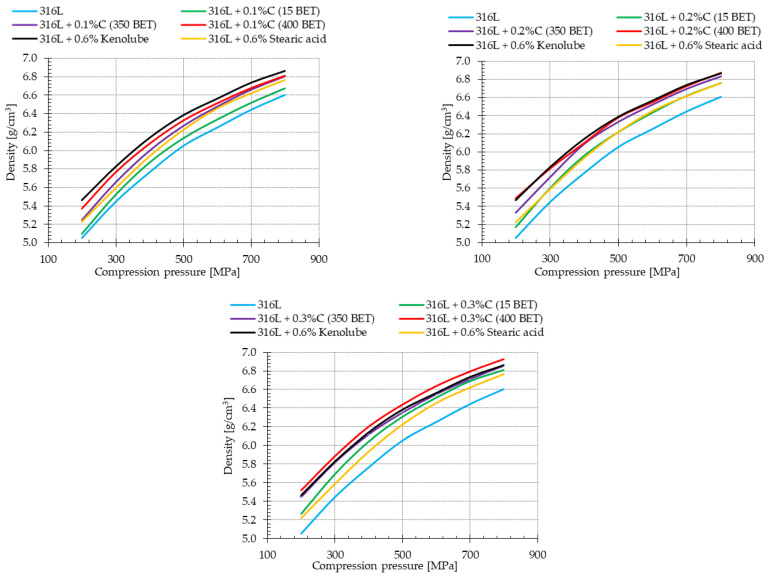
Density curves for pure powder of AISI 316L austenitic stainless steel and mixtures of this powder with additions of graphite powders and slip agents.

Powder compaction is a significant parameter in determining the ability to form a shape with stable and defined dimensions. For this study, density curves were prepared for the investigated pure stainless steel powder and mixtures of this steel with micrographite, nanographite powders addition, and with the addition of the lubricants stearic acid and Kenolube (Figure 2).

The analysis of the density curves shows that, for all the mixtures, the density of compacted products increased with the increasing value of the pressure applied during pressing. The pure powder of AISI 316L austenitic stainless steel had the lowest density. The applied additives of micrographite and nanographite powders, and the lubricant additives, allowed for an increase in the densities of the compacts. Analyzing the obtained results, it can be observed that the density of the compacts, for all the applied pressing pressures, increased with an increase in the BET specific surface area and the percentage of micro- and nanographite in the mixtures. The highest density was observed for the samples made of basic powder with 0.3 wt% nanographite with 400 m^2^/g BET addition. Equally, high densities, practically at the same level, were characteristic of AISI 316L austenitic stainless steel powder blends with 0.2 wt% of graphite nanopowder with 350 m^2^/g BET and 0.6% by weight of Kenolube. The density of the mixture with the addition of 0.6 wt% stearic acid practically coincides with the results obtained for the blend with the addition of 0.2 wt% graphite micropowder. The analysis of the results concludes that the use of graphite nanopowders as an additive modifying the powder of AISI 316L austenitic stainless steel positively influences its density.

In powder metallurgy, the sintering process aims to remove the lubricants altogether. Therefore, it should be remembered that it is inadvisable to use high pressures during pressing, which results in a decrease in the proportion of open porosity, hindering the removal of lubricants. Considering the above fact, and the recommendations provided in the material sheets of the tested powders, a pressing pressure of 600 MPa was used for further testing. This pressure is considered, in this case, to be the most favorable in terms of contribution of the obtained open porosity, as a function of the sintered density.

### 3.2. The Ejection Force While Removing the Compacts from the Die

**Figure 3:**
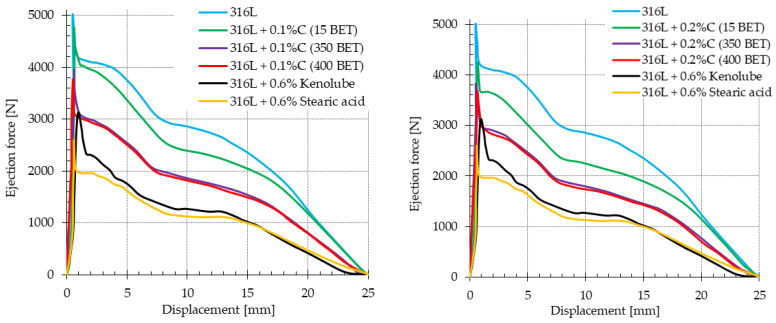

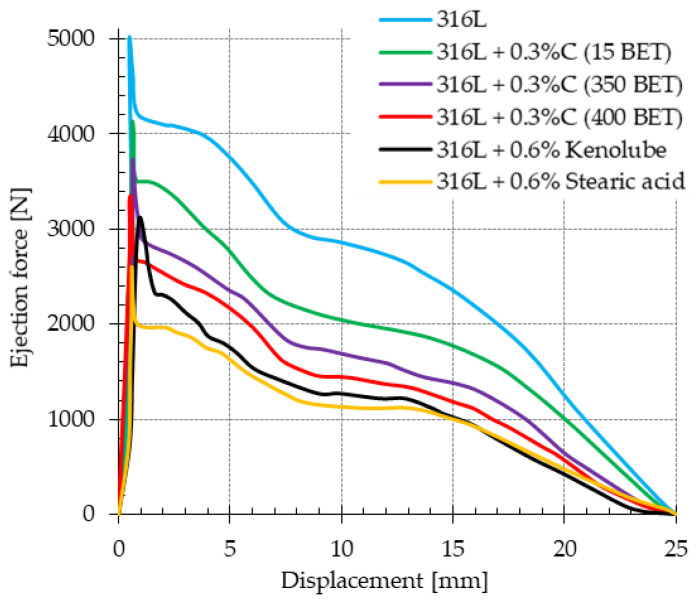
Dependence of the ejection force from the die as a function of the punch displacement for pure powder of AISI 316L austenitic stainless steel and mixtures of this powder with additives of graphite powders and lubricants.

Figure 3 shows the forces of ejection of the moldings from the die, depending on the additives introduced into the powder of AISI 316L austenitic stainless steel-micrographite and nanographite powders, as well as the additives of lubricants. The ejection of the moldings from the die took a typical course for all the tested samples, differentiated concerning their composition. The appearance of a local maximum on the curves, with a sudden drop, is a result of the start of the movement of the punch and the molded part, as well as the breaking of bonds formed during pressing between the die walls and the ejected sample. The following small jump in the value of the ejection force, visible on the obtained curves, is probably caused by the loosening of the lower punch in the die. In the last stage of the ejection process, the value of the ejection force reaches zero.

Table 4 presents the obtained values of maximum force while removing the compacts from the die, depending on the percentage composition of the mixtures. Analysis of the obtained results showed that both the amounts and type of additives have a significant effect on the value of the ejection force of the molded part from the die. The value of the maximum ejection force decreases with an increase in the content of introduced graphite powders. In addition, the degree of the specific surface area development of graphite particles is not insignificant; as it increases, the maximum value of the force required to push the compact out of the die decreases. Among the samples made of mixtures with added graphite micro- and nanopowders, the lowest maximum values of force for pushing out the moldings from the matrix were characterized by those with 0.2 wt% and 0.3 wt% of nanographite powder with 400 m^2^/g BET. Their values were 27% and 33% lower, respectively, than the value recorded for the sample made of basic powder under study. The lowest ejection force values were recorded for samples made of AISI 316L steel doped with 0.6 wt% stearic acid and Kenolube; the maximum ejection force for these mixtures was about 37% and 48% lower, respectively, compared to the obtained value for the compacts made of the basic powder.

Simchi [19], Li [43], and Wikman [44], in their research work, demonstrated that the coefficient of friction (a measure of the frictional interactions occurring between the matrix walls and the powder particles) decreases after the addition of lubricants. At the same time, different types of lubricants produce other lubrication effects. Rażniewska, in her work, studied the impact of lubricants based on amide waxes, palmitic acid, stearic acid, lithium stearate, and lauryl new acid on the process of aluminum powder pressing. These studies showed that the best result—the lowest value of the ejection force of the molded part from the die—was obtained when the die walls were lubricated with lubricants based on amide waxes [45]. On the other hand, Nia and Davies found an increase in the density of aluminum powder pressed using Nopocowax (a mixture of amides), Acrawax, and tin stearate [46]. In addition, the research results presented in Simchi [19,27] and Bergkvist’s [47] works indicate that improper selection of a lubricant can result in increased wear of die and parts with poor surface finish.

Mares and Tamashausky [29], in their work, conducted a study using nanographite powders, derived from fully graphitized raw materials, to determine if nanographite materials can be used instead of, or to enhance, conventional lubricants used in PM. The authors focused on determining the suitability of nanographite materials to provide adequate matrix wall lubrication, reducing or eliminating the need to add lubricants. The results of their study showed that, compared to conventional graphite micropowder, nanographite powders provide significant improvements in die wall lubricity and exhibit lubrication synergy with powder mixtures containing reduced levels of slip agent, in addition to delivering metallurgically active carbon during the sintering process.

### 3.3. Density and Porosity

**Figure 4:**
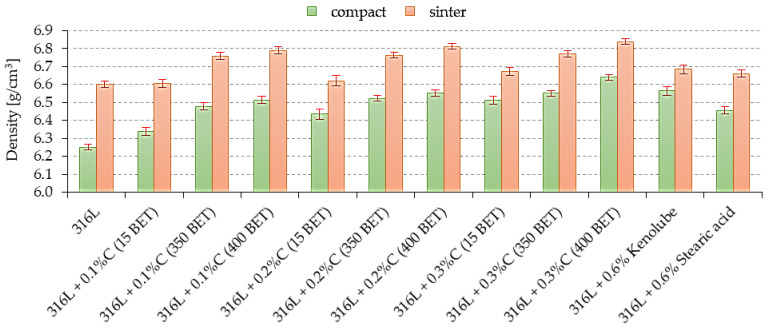
The compacts and sinters densities depend on the percentage composition of mixtures.

Figure 4 shows example results of density for compacts and sinters made of the prepared mixtures based on tested AISI 316L austenitic stainless steel with micrographite and nanographite powders, as well as the lubricants stearic acid and Kenolube. The test specimens were pressed in a hydraulic press at 600 MPa.

Both the amount and type of additives have a significant effect on the value of the measured densities of tested compacts and sinters. Samples with graphite additive showed the following tendency; the higher the percentage amount of graphite additive in the mixture, and the more developed the BET specific surface area of graphite particles, the higher the obtained density values, for both compacts and sinters, were. In addition, the use of lubricants—stearic acid and Kenolube—increased the density of compacts and sinters compared to the values obtained for samples made of pure AISI 316L powder. The highest density was obtained for samples with the 0.3 wt% addition of nanographite with a 400 m^2^/g BET.

**Figure 5:**
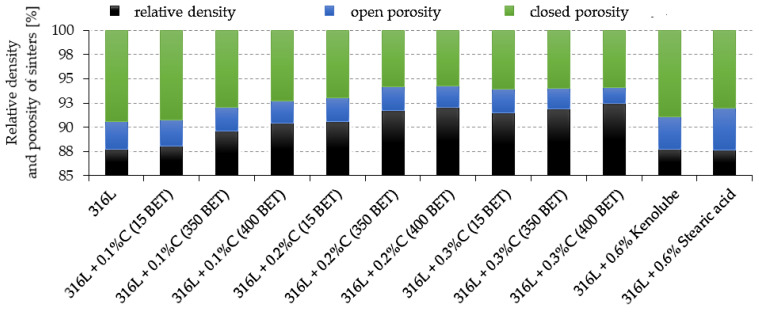
The relative densities and porosity of the sinters depend on the percentage composition of mixtures.

Figure 5 shows the results of relative density and porosity measurements for sinters. The type of additive used and its amount in the mixture significantly affected the obtained values of relative density and porosity. The dominant kind of porosity for tested sinters is closed porosity. An increase in the amount of added graphite micro- and nanopowders, as well as the degree of development of the BET specific surface area of the graphite particles, contributes to a decrease in the open porosity and total porosity of the tested sinters.

Sinters made from blends with 0.2 wt% and 0.3 wt% nanographite, with 400 m^2^/g BET, have the lowest total porosity, about 8% and 7.5%, respectively. The sinters made from the blend with the addition of Kenolube lubricant had a similar total porosity as those made from pure austenitic stainless steel powder, with slightly higher open porosity. On the other hand, the sinters of the studied steel doped with stearic acid were characterized by the highest level of total porosity, as well as the highest proportion of the open porosity type—about 12.5% and 4.5%, respectively. Such a high value of total porosity can negatively affect the mechanical properties and corrosion resistance of the sinters.

As shown in previous studies [35], the addition of graphite acts as an activator during the sintering process in a vacuum of AISI 316L austenitic stainless steel; it contributes to the intensification of the reduction reaction of the oxide layer on the tested steel particles surface. Therefore, samples with the addition of graphite sintered better and achieved higher density values and a lower share of total porosity.

For comparison, the research conducted by S. Ali et al. [48] on austenitic stainless steel with the addition of 0.25% boron, sintered at the temperature of 1200 °C, showed the achievement of 89.5% relative density. Moreover, it should be remembered that the addition of boron causes the formation of brittle boron silicide during the sintering process.

### 3.4. Microstructure

**Figure 6:**
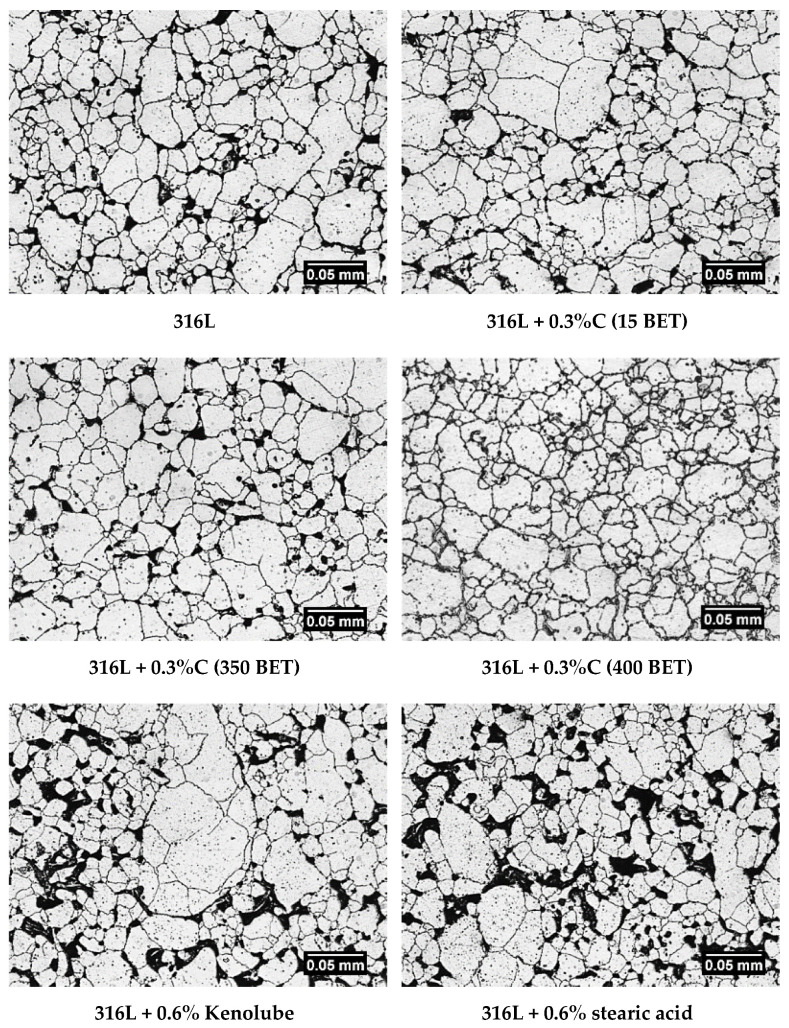
Example microphotographs of etched metallographic specimen surfaces showing the microstructure of sinters made from pure AISI 316L austenitic stainless steel powder and mixtures with the addition of 0.3 wt% of micrographite (15 m^2^/g BET), nanographite (350 m^2^/g and 400 m^2^/g), 0.6 wt% of Kenolube, and 0.6 wt% of stearic acid.

Figure 6 shows the microstructure of selected tested sintered stainless steels in the etched state. All the microphotographs show clear grain boundaries. Based on the surface microphotographs of all the tested materials, it can be concluded that the tested sintered stainless steels had a single-phase microstructure—austenitic. Moreover, it was observed that, with the increasing content of graphite powder introduced into 316L steel and the BET specific surface area of its particles, there is a slight decrease in grain size. Analyzing all the microphotographs obtained, it can be concluded that, with an increase in the amount and more developed the BET specific surface area of graphite particles introduced into 316L steel, both the amount and the pore size decreased. On the other hand, stainless steel doped with lubricants—Kenolube and stearic acid—was characterized by a high proportion of irregular pores of significant size.

### 3.5. Mechanical Properties

To determine the mechanical properties of the sintered materials, a series of tests were carried out, including a static tensile test, Kc impact test, three-point bending test, Rockwell hardness measurements, and Vickers microhardness measurements.

The values of Young’s modulus (E), tensile strength (R_m_), conventional yield strength (R_0.2_), and relative percentage elongation (A_5_), determined from the analysis of the curves obtained from the static tensile test, are summarized, along with other mechanical properties, in Table 5.

Table 5 summarizes the values of all tested mechanical properties. On the basis of the analysis of the obtained test results, it can be concluded that the mechanical properties of the tested sinters significantly depend on the amount, as well as the type, of graphite additive; with an increase in the weight fraction, as well as the development of the BET specific surface area of graphite powder particles introduced as an additive into the powder of AISI 316L austenitic stainless steel, a significant improvement in the mechanical properties of the tested sinters is observed. In summary, the sinters made of base powder with additives of 0.2 wt% and 0.3 wt% of nanographite powder with 400 m^2^/g BET had the highest mechanical properties—they are marked in green in the table. In contrast, the addition of slip agents did not improve the mechanical properties of the tested steel.

**Figure 7:**
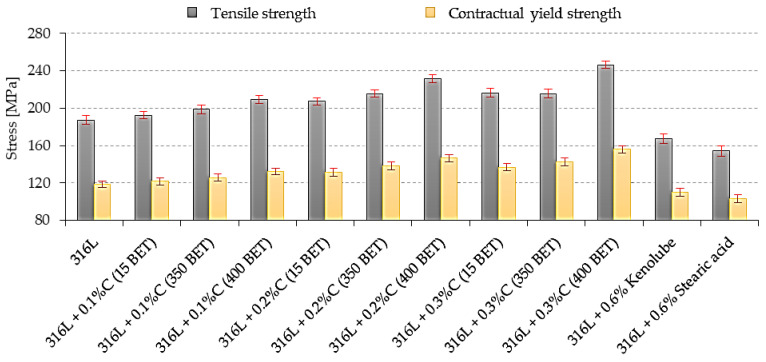
The tensile strength and contractual yield strength of sinters depends on the percentage composition of mixtures.

**Figure 8:**
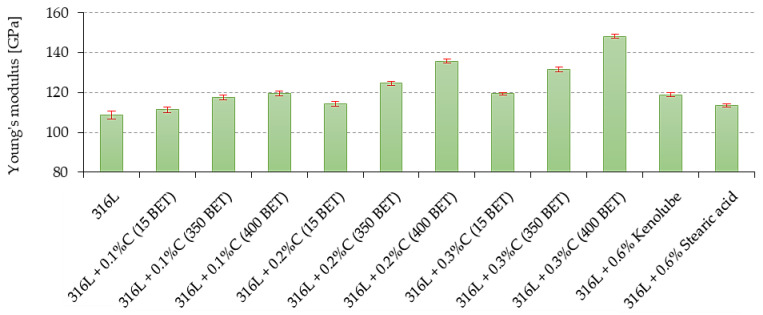
Young’s modulus of sinters depends on the percentage composition of mixtures.

Figure 7 summarizes the tensile strength and contractual yield strength values, along with the standard deviations determined for all the sinters tested. Figure 8 shows Young’s modulus values for all the tested sinters. The addition of graphite, in the form of micro- and nanopowder, resulted in an increase in Young’s modulus, tensile strength, and contractual yield strength, with a simultaneous improvement in the plastic properties of the sinters; this increase was higher the larger the percentage amount of graphite additive in the mixture, and the more developed the BET specific surface area of the graphite particles.

The highest values of Young’s modulus, tensile strength, conventional yield strength, and relative elongation were observed for sinters made of mixtures with 0.2 wt% and 0.3 wt% of nanographite with 400 m^2^/g BET. The 0.2 wt% and 0.3 wt% additions of this nanographite powder increased the values of the discussed properties by about 25% and more than 30%, respectively, in comparison to the values obtained for sinters made of pure AISI 316L powder. The addition of lubricants to the AISI 316L steel resulted in a deterioration of its strength and plastic properties, which was probably due to the higher porosity of these sinters.

**Figure 9:**
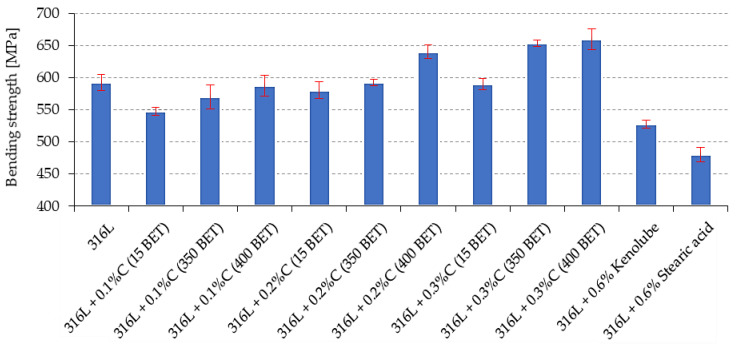
The bending strength of sinters depends on the percentage composition of mixtures.

**Figure 10:**
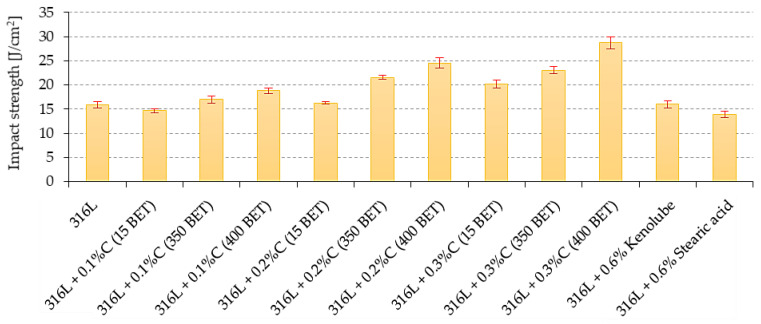
The impact strength of sinters depends on the percentage composition of mixtures.

For the results obtained from the three-point bending test and the impact strength measurements, the trends observed were similar to those obtained from the static tensile test. The flexural strength (Figure 9) and impact strength (Figure 10) of the sintered materials increased with an increase in the weight fraction and the development of the BET specific surface area of the graphite powder particles. The contribution of 0.2 wt% and 0.3 wt%, by weight, of nanographite powder with 400 m^2^/g BET, introduced into the AISI 316L austenitic stainless steel powder, allowed an increase of the bending strength by about 10.5% and 11.5%, respectively, and of the impact strength by about 63% and 87%, respectively, relative to the values obtained for the reference sinters made of pure AISI 316L powder. In the case of sinters made of mixtures with the addition of lubricants, a reduction in the values of the tested properties concerning the reference sample is observed.

**Figure 11:**
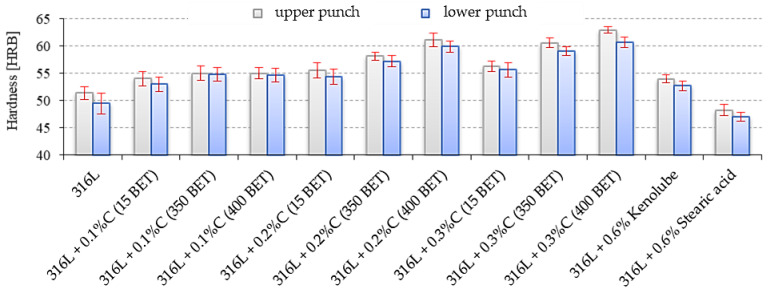
Rockwell hardness B scale of sinter, measured from the upper and lower punch side, depending on the percentage composition of the tested mixtures.

HRB hardness measurements were carried out on the sinter surface from the side of the upper (pressing) punch and the side of the lower punch (Figure 11). Analyzing the results obtained, it can be observed that the increase in the hardness of sintered particles depended both on graphite content and the BET specific surface area of its particles; the more developed the specific surface area of graphite powder added to austenitic stainless steel powder, the higher the values of sinter hardness obtained. The increase in sinter hardness was probably due to the increased compaction and more favorable pore morphology (greater spheroidization of pores and their reduced size) resulting from the intensified sintering process [35].

**Figure 12:**
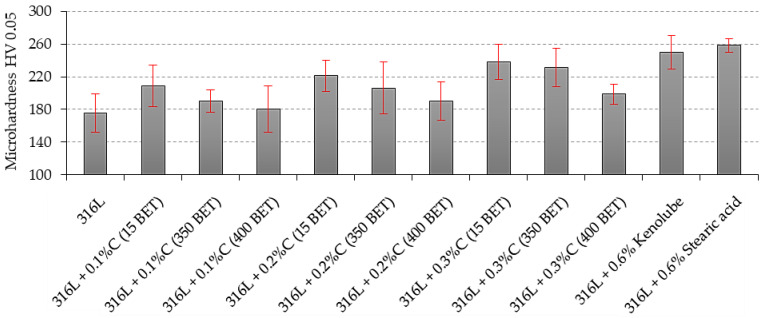
Rockwell hardness B scale of sinter, measured from the side of the upper and lower punch, depending on the percentage composition of the tested mixtures.

In the case of microhardness of the tested sinters, a different trend was observed (Figure 12). A decrease in the measured values can be observed for samples made of mixtures with the nanographite addition with a more developed BET specific surface area, which does not change the fact that these values were higher than those obtained for sinters made of pure AISI 316L powder. For sinters made of the tested stainless steel with the addition of graphite, the microhardness values ranged from about 180 to about 240 HV. These results showed a close correlation with the measured carbon content in the samples after sintering, the results of which were published in a previous paper [35]. While the hardness of the sinter mainly depended on its porosity, in the case of microhardness, its value was influenced primarily by the content of dissolved carbon in the grain. Due to the specificity of sinter microhardness measurements, the calculated standard deviations were relatively large.

For comparison, S. Ali et al. [48], in their study, obtained a microhardness of 189 HV after adding 0.25% boron to austenitic stainless steel. A slightly lower value of microhardness for austenitic 316L stainless steel sintered in an argon atmosphere at 1300 °C with a soaking time of 30 min, equal to 181 HV, was achieved by Kurgan [49] in his research.

**Figure 13:**
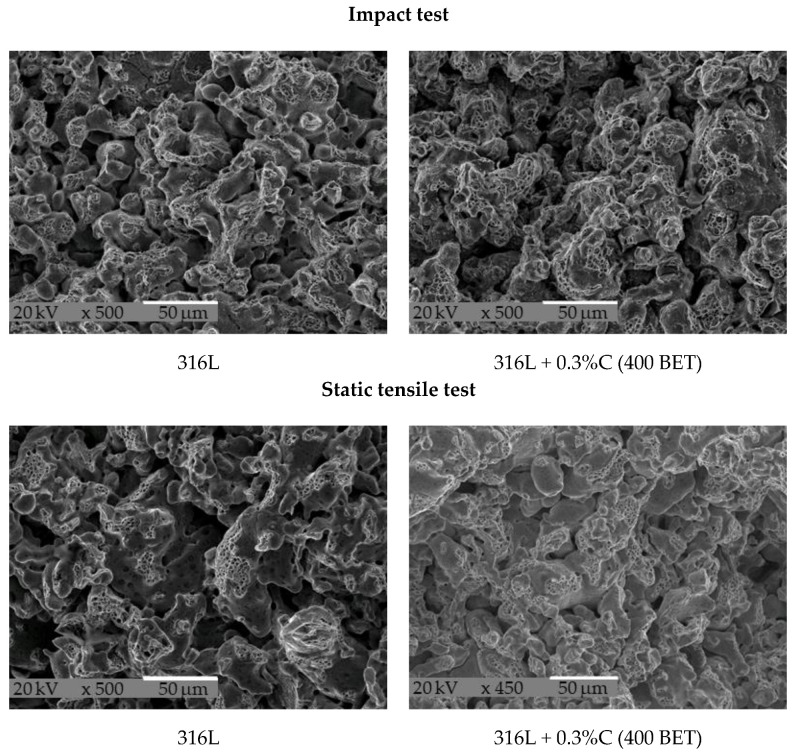

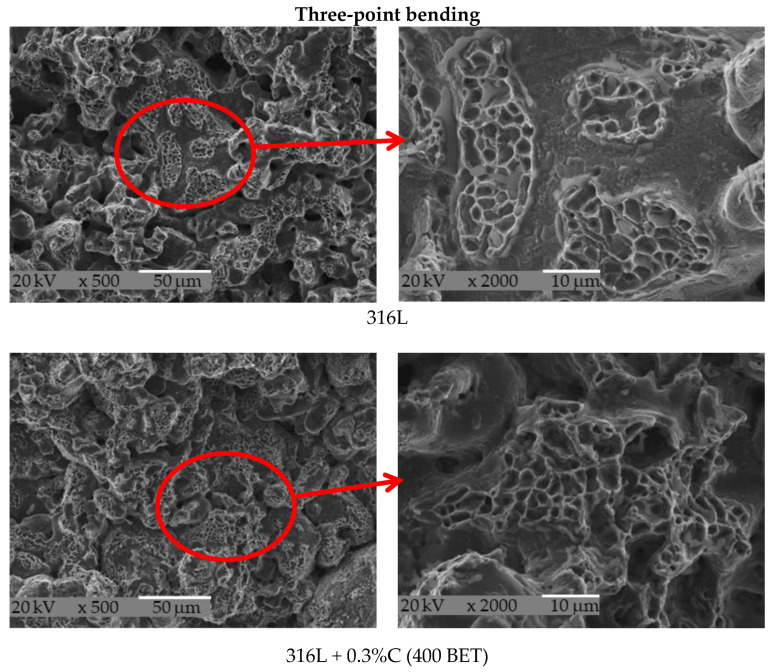
Topography of fractures of sintered AISI 316L austenitic stainless steel powder with the addition of 0.3 wt% of nanographite powder with 400 m^2^/g BET after impact test, static tensile test, and three-point bending test.

Fractographic analysis for all tested materials was performed on impact specimens, broken at 20 °C. Fracture topography was also observed on specimens from static tensile tests and three-point bending. Photographs of the fractures were taken using a JOEL JSM5510LV scanning electron microscope. Figure 13 shows an example of the fractographic pictures of the fractures obtained from the impact test, static tensile test, and three-point bending test, respectively, for a specimen made of tested steel powder and its mixture with 0.3 wt% nanographite powder with 400 m^2^/g BET.

Based on the observation of all the fracture photographs, it was found that the fracture surface topography of all the tested samples exhibited the characteristics of transcrystalline ductile fracture. The applied additives in graphite micro- and nanopowders and lubricants did not cause any apparent changes in the very nature of the observed fractures. However, as the density of the samples increases, the development of the fracture surface intensifies, and the number of microvoids in the area of sinter necks, which are characteristic of ductile fracture, increases during the sintering process of the investigated steels. Most of these voids have a diameter not exceeding 2 μm. In the case of sintering made from a mixture of base powder with the addition of lubricants, due to their high porosity, a more significant number of smooth surfaces—which were the walls of pores, not constituting the separating surfaces of the material—and an increased number of voids can be observed on the surface of the fractures of the tested samples.

**Figure 14:**
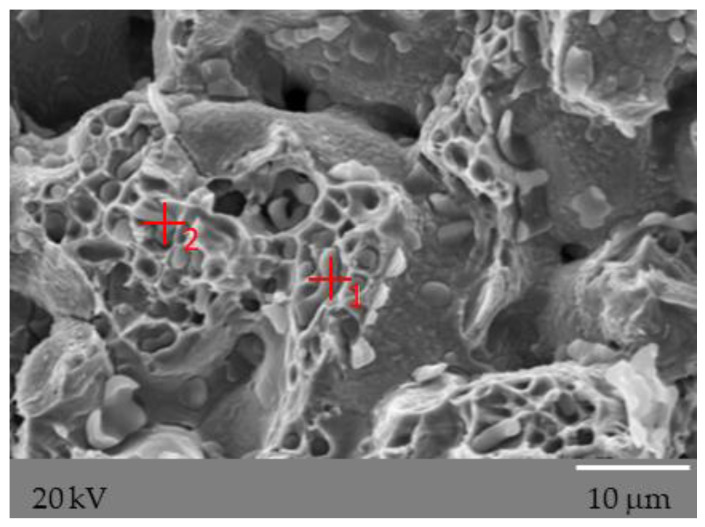
The oxides observed at breakthrough from three-point bending of a sinter made of AISI 316L austenitic stainless steel powder with 0.3 wt% graphite nanopowder with a BET specific surface area of 400 m^2^/g.

**Figure 15:**
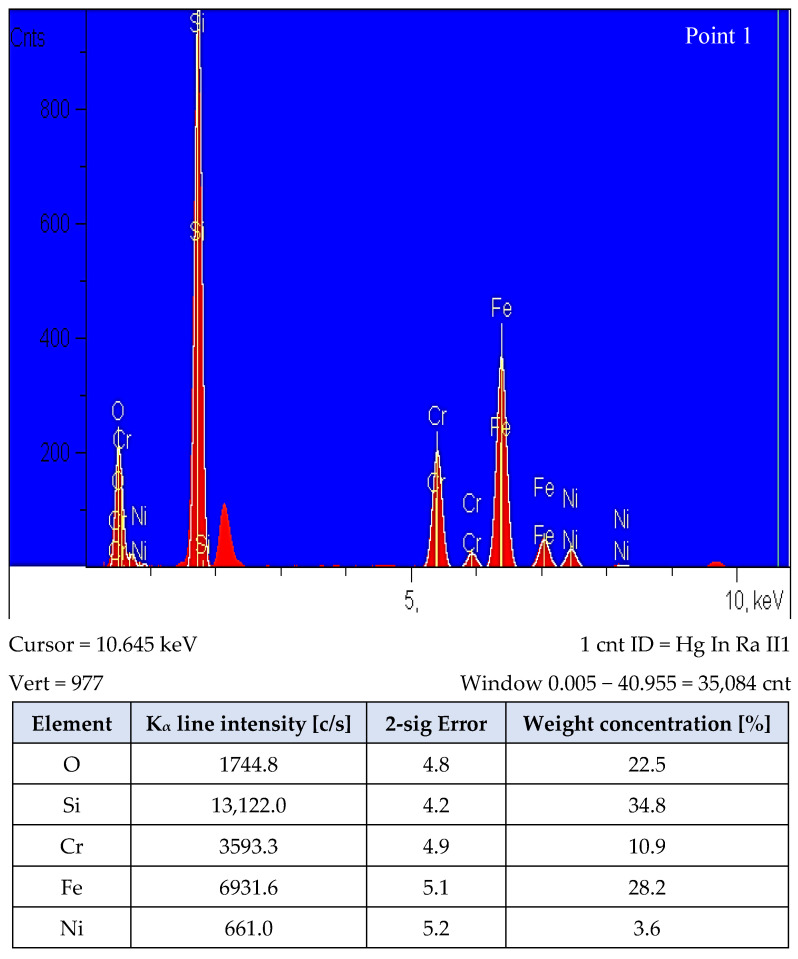

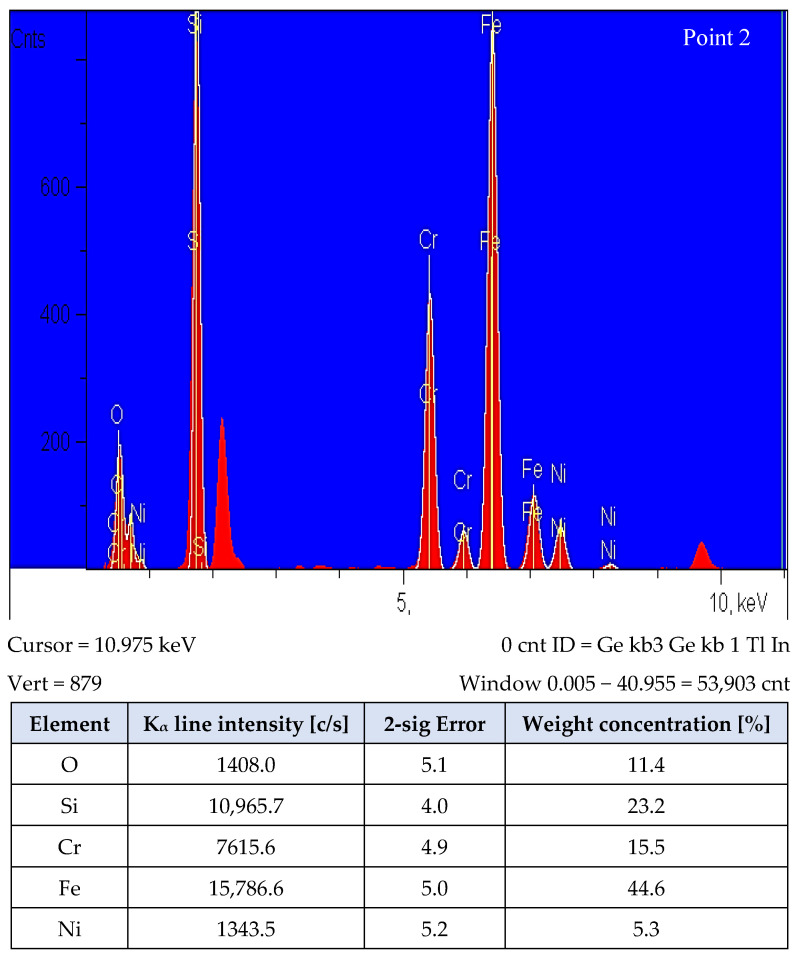
The energy spectrum and microanalysis results of the observed oxides’ chemical composition at the breakthrough from three-point bending of a sinter made of AISI 316L austenitic stainless steel with 0.3 wt% graphite nanopowder with a BET specific surface area of 400 m^2^/g.

Photographs of all analyzed fractures show single, irregular separations, significantly different from the observed surface. However, these separations, due to their small size, are only visible at very high magnifications (Figure 14). The EDS analysis carried out on a scanning electron microscope (Figure 15) allowed the identification of these precipitates as complex oxides. Similar research results were presented by Castro and Lozada [50], who explained the occurrence of these precipitates, which are complex oxides with high silicon content, as a residue of the oxide surface covering the powder particles before the sintering process. The obtained results of the EDS analysis constitute only a qualitative analysis of the elements present, due to the measurement inaccuracy (high degree of dispersion) resulting from the uneven surface of the studied fractures.

## 4. Conclusions

In this manuscript, the effect of AISI 316L austenitic stainless steel modification with the addition of micro- and nanographite powders and lubricants on the pressing process and selected mechanical properties was investigated. The analysis of the obtained research results allowed us to formulate the following conclusions:

- Graphite nanopowders provide improved powder densities of AISI 316L austenitic stainless steel. The increase in densification is more significant the greater the proportion of graphite powder and the degree of BET specific surface area development of its particles. With a greater degree of surface, development comes a reduction in the particle size of the graphite used in the tests. As a result, the finer particles fill the spaces between the steel powder particles to a greater extent, producing a lubricating film on their surfaces during the powder mixing stage. During the pressing process, this provides better lubrication between the steel powder particles in contact and between the particles and the die walls.
- The reduction in friction between the pressed powder and the die walls allows the molded part to be pushed out of the die with significantly less force, ultimately leading to a reduction in die wear. The lowest values of the maximum ejection force of the moldings from the die for the blends of the tested steel doped with graphite powders were achieved for the additions of 0.2 wt% and 0.3 wt% of nanographite powder with 400 m^2^/g BET. The ejection force values for these blends were similar to the lowest ones obtained for mixtures of AISI 316L austenitic stainless steel powder with Kenolube and stearic acid additives.
- Graphite micropowder does not allow such a significant improvement in densification as graphite nanopowder provides. This is most likely due to the larger size of its particles, which are not able to fill the spaces between the powder particles of the tested steel to the same extent as the finer particles of nanographite. In addition, for this reason, the graphite micropowder does not provide as beneficial lubricating properties as the nanographite powder when ejecting the molded part out of the matrix.
- The sintering agents fulfill their role; they significantly improve the densification of AISI 316L steel powders and decrease the forces of ejection of the moldings out of the die during the pressing process. However, the results obtained in this study indicate that the use of stearic acid and Kenolube as additives in the powder of the stainless steel understudy adversely affects the degree of sinter thickening and the pore morphology after sintering. The formation of large irregularly shaped pores is probably due to the impeded evacuation of the gaseous products of the thermal decomposition of the lubricants outside the sintered sample. This results in a decrease in the mechanical properties and the corrosion resistance of the sinter.
- Based on the above, it can be concluded that the addition of graphite nanopowder provides the possibility to press the investigated AISI 316L steel powder without the need to use commercially available slip agents. Consequently, it allows for shortening of the duration of the sintering process by eliminating the firing step of these agents, which can be a clear economic advantage from a practical point of view.
- The addition of nanographite allows for a significant increase in such mechanical properties as tensile strength, contractive yield strength, relative elongation, flexural strength, impact strength, hardness, and microhardness of sintered AISI 316L austenitic stainless steel. The higher the percentage amount of graphite additive in the mixture and the more developed the BET specific surface area of graphite particles, the better the properties of the tested steel. The addition of nanographite, in comparison to graphite micropowders, provides better reducing properties during the sintering process, resulting in an increase in the degree of sinter compaction and changes in pore morphology (spheroidization and decrease in pore number). This contributes to improving the discussed properties of sintered AISI 316L stainless steel.

## Figures and Tables

**Table 1:** Chemical composition of AISI 316L austenitic stainless steel powder [35].

Chemical Element	C	Ni	Cr	Mn	Si	N	S	Mo	O *	Fe
Percentage by weight (% wt.)	0.018	12.9	17.0	0.1	0.9	0.06	0.01	2.3	0.14	Bal.

* Oxygen occurs as an oxide layer on the surface of powder particles (defined according to PN-EN ISO 4491-2:2002 [36]).

**Table 2:** Characteristics of the lubricants used in the study.

Lubricant	Form of the Solid-State at Ambient Temp.	Melting Point (°C)	Flashpoint (°C)	Auto-Ignition Temperature (°C)	Density (g/cm^3^)
Stearic acid	powder	67	196	395	0.94
Kenolube P11	powder	100~140	-	-	1.054

**Table 3:** The composition of the individual tested mixtures and the adopted designation.

No.	Composition of the Mixture (% wt.)	Designation of the Sample
1	100% AISI 316L	316 L
2	99.9% AISI 316L + 0.1% micrographite (15 m^2^/g BET)	316L + 0.1%C (15 BET)
3	99.8% AISI 316L + 0.2% micrographite (15 m^2^/g BET)	316L + 0.2%C (15 BET)
4	99.7% AISI 316L + 0.3% micrographite (15 m^2^/g BET)	316L + 0.3%C (15 BET)
5	99.9% AISI 316L + 0.1% nanographite (350 m^2^/g BET)	316L + 0.1%C (350 BET)
6	99.8% AISI 316L + 0.2% nanographite (350 m^2^/g BET)	316L + 0.2%C (350 BET)
7	99.7% AISI 316L + 0.3% nanographite (350 m^2^/g BET)	316L + 0.3%C (350 BET)
8	99.9% AISI 316L + 0.1% nanographite (400 m^2^/g BET)	316L + 0.1%C (400 BET)
9	99.8% AISI 316L + 0.2% nanographite (400 m^2^/g BET)	316L + 0.2%C (400 BET)
10	99.7% AISI 316L + 0.3% nanographite (400 m^2^/g BET)	316L + 0.3%C (400 BET)
11	99.4% AISI 316L + 0.6% Kenolube	316L + 0.6% Kenolube
12	99.4% AISI 316L + 0.6% stearic acid	316L + 0.6% Stearic acid

**Table 4:** Values of the maximum force while removing the compacts from the die depend on the percentage composition of mixtures.

Scheme	Maximum Ejection Force (N)
316L	4991 ± 4.79
316L + 0.1%C (15 BET)	4726 ± 5.32
316L + 0.1%C (350 BET)	3930 ± 5.75
316L + 0.1%C (400 BET)	3766 ± 6.21
316L + 0.2%C (15 BET)	4252 ± 5.75
316L + 0.2%C (350 BET)	3819 ± 4.82
316L + 0.2%C (400 BET)	3660 ± 5.75
316L + 0.3%C (15 BET)	4143 ± 5.23
316L + 0.3%C (350 BET)	3713 ± 4.94
316L + 0.3%C (400 BET)	3339 ± 5.67
316L + 0.6% Kenolube	3135 ± 7.88
316L + 0.6% stearic acid	2611 ± 5.44

**Table 5:** The mechanical properties of sinters depend on the percentage composition of the mixtures.

Sample Determination	Total Porosity P_c_ (%)	Static Tensile Test				Three-Point Bending Test		Impact Strength Kc (J/cm^2^)	Hardness	
		Tensile Strength R_m_ (MPa)	Contractual Yield Strength R_0.2_ (Mpa)	Relative Elongation Percentage A_5_ (%)	Young’s Modulus E (Gpa)	Bending Strength σ_g_ (Mpa)	Deformation at Failure ɛ_z_ (%)		HRB	HV
316L	12.27	186 ± 4.3	119 ± 3.9	4.79 ± 0.12	108 ± 2.1	593 ± 13.2	7.4 ± 0.005	15 ± 0.8	51 ± 1.3	100 ± 1.3
316L + 0.1%C (15 BET)	11.88	190 ± 3.9	123 ± 4.1	4.95 ± 0.19	110 ± 1.7	547 ± 7.2	6.4 ± 0.007	14 ± 0.6	54 ± 1.5	103 ± 1.5
316L + 0.1%C (350 BET)	10.33	199 ± 4.3	125 ± 3.8	5.02 ± 0.15	119 ± 1.2	572 ± 13.5	7.6 ± 0.011	18 ± 0.7	54 ± 1.4	103 ± 1.2
316L + 0.1%C (400 BET)	9.56	210 ±4.7	133 ± 4.2	5.18 ± 0.17	120 ± 1.9	589 ± 14.3	7.3 ± 0.004	20 ± 0.4	56 ± 01.0	105 ± 1.5
316L + 0.2%C (15 BET)	9.42	205 ± 4.5	132 ± 3.7	5.10 ± 0.18	112 ± 1.4	579 ± 16.7	6.7 ± 0.008	15 ± 0.6	57 ± 1.1	106 ± 1.1
316L + 0.2%C (350 BET)	8.27	216 ± 4.7	139 ± 4.3	5.45 ± 0.22	123 ± 1.9	593 ± 6.8	7.1 ± 0.003	23 ± 0.4	59 ± 0.9	107 ± 0.9
316L + 0.2%C (400 BET)	7.97	233 ± 3.9	148 ± 3.2	5.95 ± 0.19	135 ± 1.2	655 ± 7.2	7.7 ± 0.009	26 ± 0.8	61 ± 1.6	110 ± 1.1
316L + 0.3%C (15 BET)	8.47	211 ± 4.4	137 ± 4.1	5.19 ± 0.23	120 ± 2.0	589 ± 11.2	6.9 ± 0.007	19 ± 0.7	56 ± 0.9	105 ± 0.9
316L + 0.3%C (350 BET)	8.11	218 ± 4.1	142 ± 4.4	5.70 ± 0.15	134 ± 1.9	643 ± 9.5	7.2 ± 0.08	23 ± 0.6	61 ± 0.1.3	110 ± 1.3
316L + 0.3%C (400 BET)	7.47	245 ± 4.2	155 ± 3.5	6.30 ± 0.21	149 ± 1.5	661 ±08.9	8.3 ± 0.010	30 ± 0.9	63 ± 0.9	113 ± 0.8
316L + 0.6% Kenolube	12.28	165 ± 4.7	109 ± 4.2	4.13 ± 0.21	120 ± 1.7	526 ± 7.6	7.0 ± 0.008	15 ± 0.5	54 ± 1.2	103 ± 1.3
316L + 0.6% Stearic acid	12.33	155 ± 4.9	104 ± 4.1	3.89 ± 0.20	112 ± 1.6	479 ± 8.5	4.6 ± 0.011	13 ± 0.7	48 ± 1.1	97 ± 1.4

## Data Availability

Not applicable.

## References

[1] Molinari A., Cristofolini I., Marchetti F., Tiziani A. (1992). Auger electron spectroscopy study of martensitic stainless steel sintered in different atmospheres. Powder Metall. Int..

[2] Kazior J. (1994). Analiza Czynników Technologicznych Decydujących o Własnościach Spiekanych Austenitycznych Stali Nierdzewnych.

[3] Molinari A., Kazior J., Marchetti F., Cantieri R., Cristofolini I., Tiziani A. (1994). Sintering mechanisms of Boron alloyed AISI 316L stainless steel. Powder Metall..

[4] Terrisse C., Nyborg L., Bracconi P. Surface reactions during vacuum sintering of 316L stainless steel powder. Proceedings of the 1998 Powder Metallurgy World Congress and Exhibition.

[5] Tunberg T., Nyborg L., Liu C.X. (1992). Enhanced vacuum sintering of water-atomized austenitic stainless steel powder by carbon addition. Adv. Powder Metall. Part. Mater..

[6] Toennes C., German R.M. (1992). Density and microstructure control in a martensitic stainless steel through enhanced sintering. Powder Metall. Int..

[7] Skałoń M., Kazior J. (2012). Enhanced sintering of austenitic stainless steel powder AISI 316L through boron containig master alloy addition. Arch. Metall. Mater..

[8] Larsen R.M., Thorsen K.A. (1994). Equilibria and kinetics of gas-metal reactions during sintering of austenitic stainless steel. Powder Metall..

[9] Danninger H., Nikolov D., Leitner G., Jaenicke-Robler K. Deoxidation processes during sintering of steel compacts prepared from pre-oxidized iron and steel powders. Proceedings of the International Conference “Sintering ‘03”.

[10] Lindqvist B. Chromium alloyed steels—A new powder generation. Proceedings of the Euro PM 2001 Congress and Exhibition.

[11] Larsen R.M., Thorsen K.A. Influence of sintering atmosphere on corrosion resistance and mechanical properties of sintered stainless steel. Proceedings of the International Conference on Materials by Powder Technology (PTM-93).

[12] Pao M.A., Klar E. (1983). Corrosion phenomena in regular and tin modified P/M stainless steel. Prog. Powder Metall..

[13] Beste U., Sundin S., Petrini D. The role of oxygen content on properties of PM materials. Proceedings of the International Conference on Powder Metallurgy and Particulate Materials.

[14] Buscali H., El Messki S., Riffard F., Perrier S., Cueff R., Issartel C. (2008). Role of molybdenum on the AISI 316L oxidation at 900 °C. J. Mater. Sci..

[15] Bergman O., Frisk K., Nyborg L. Analysis of oxide reduction during sintering of Cr-alloyed steel powder through Photoacoustic Spectroscopy Meassurements. Proceedings of the Euro PM2009 Congress and Exhibition.

[16] Danninger H., Wolfsgruber E., Ratzi R. Gas Formation during sintering of PM steels containing carbon. Proceedings of the Euro PM97 Congress and Exhibition EURO PM.

[17] Rawlings A., Hanejko F. Die wall lubricant utilizing warm compaction methods. Proceedings of the International Conference on Powder Metallurgy and Particulate Materials, Part 3 Compaction and Forming.

[18] Rawlings A., Luk S., Hanejko F. (2000). Engineered Approach to High Density Forming Using Internal and External Lubricants.

[19] Simchi A., Nojoomi A.A. (2013). Warm compaction of metallic powders. Advances in Powder Metallurgy: Properties, Processing and Applications.

[20] Feng S.S., Geng H.R., Guo Z.Q. (2012). Effect of lubricants on warm compaction process of Cu-based composite. Compos. Part B.

[21] Ngai T.L., Chen W.P., Xiao Z. (2002). Die wall lubricated warm compaction of iron-based powder metallurgy material. Trans. Nonferrous Metall. Soc..

[22] Barrow D.A. (1988). Developments in powder metallurgy (PM) materials. Mater. Des..

[23] Saha D., Apelian D. (2002). Control Strategy for the De-lubrication of P/M Compacts. Int. J. Powder Metall..

[24] Rodrigues H., Madill S., Folliard M., Liu T. Optimizing Compacting Lubricant Selection—A Comparison Study of Various Commercially Available Lubricants. Proceedings of the World Congress on Powder Metallurgy, Particulate Materials.

[25] St-Laurent S., Thomas Y., Azzi L. (2006). High performance lubricants for demanding PM applications. Adv. Powder Metall. Part. Mater..

[26] Żółkowski W., Czepelak M. (1988). Wpływ rodzaju środka poślizgowego na przebieg prasowania proszku aluminium i własności spieków. Metal. Proszków.

[27] Simchi A. (2003). Effects of lubrication procedure on the consolidation, sintering and microstructural features of powder compacts. Mater. Des..

[28] Rahman M.M., Nor S.S.M., Rahman H.Y. (2011). Investigation on the effect of lubrication and forming parameters to the green compact generated from iron powder through warm forming route. Mater. Des..

[29] Mares N.T., Tamashausky A.V. (2013). Nanographite materials as solidphase integral die-wall lubricants in PM. Int. J. Powder Metall..

[30] Lindskog P. (1994). Recent developments in European powder metallurgy. Met. Powder Int..

[31] Kirkhorn L., Gutnichenko O., Melnyk O., Bushlya V., Ståhl J.E. Nano graphite flakes as lubricant additive. Proceedings of the 6th Swedish Production Symposium.

[32] Shahnazar S., Bagheri S., Abd Hamid S.B. (2016). Enhancing lubricant properties by nanoparticle additives. Int. J. Hydrogen Energy.

[33] Erdemir A., Bhushan B. (2001). Solid lubricants and self-lubricating films. Modern Tribology Handbook Materials, Coatings and Industrial Applications.

[34] Brunauer S., Emmett P.H., Teller E. (1938). Adsorption of Gases in Multimolecular Layers. J. Am. Chem. Soc..

[35] Kozub B., Kazior J., Szewczyk-Nykiel A. (2020). Sintering Kinetics of Austenitic Stainless Steel AISI 316L Modified with Nanographite Particles with Highly Developed BET Specific Surface Area. Materials.

[36] (2002). Proszki Metaliczne–Oznaczenie Zawartości Tlenu Metodami Redukcyjnymi–Część 2: Ubytek Masy w Wyniku Redukcji Wodorem (Strata Wodorowa).

[37] (2001). Spiekane Materiały Metaliczne z Wyjątkiem Węglików Spiekanych–Przepuszczalne Spiekane Materiały Metaliczne–Oznaczanie Gęstości, Zawartości Oleju i Porowatości Otwartej.

[38] (2010). Spiekane Materiały Metaliczne z Wyjątkiem Węglików Spiekanych—Próbki do Próby Rozciągania.

[39] (2018). Spiekane Materiały Metaliczne z Wyjątkiem Węglików Spiekanych—Próbka bez Karbu do Próby Udarności.

[40] (2000). Spiekane Materiały Metaliczne z Wyjątkiem Węglików Spiekanych—Oznaczanie Wytrzymałości na Zginanie.

[41] (1978). Badania Wyrobów z Proszków Metali. Oznaczanie Gęstości Pozornej Twardości i Wytrzymałości na Zginanie Spiekanych Wyrobów Ciernych.

[42] (2018). Metale. Pomiar Twardości Sposobem Vickersa. Część 1: Metoda Badania.

[43] Li Y.Y., Ngai T.L., Wang S.L., Zhu M., Chen W.P. (2005). Effect of lubricant’s friction coefficient on warm compaction powder metallurgy. Trans. Nonferrous Met. Soc. China.

[44] Wikman B., Solimannezhad N., Larsson R., Oldenburg M., Häggblad H.A. (2000). Wall Friction Coefficient Estimation through Modelling of Powder Die Pressing Experiment. Powder Metall..

[45] Rażniewska M. Gleitmittel in herstell von aluminium P/M teilen. Proceedings of the International Conference of Powder Metallurgy.

[46] Nia F.F., Davies B.L. (1982). Production of Al–Cu and Al–Cu–Si Alloys by PM Methods. Powder Metall..

[47] Bergkvist A. (2002). Warm Copaction of Steel Powders. Sweden Patent.

[48] Ali A., Rani A.M.A., Altaf K., Baig Z. (2018). Investigation of Boron addition and compaction pressure on the compactibility, densification and microhardness of 316L stainless steel. J. Mater. Sci. Eng..

[49] Kurgan N. (2013). Effects of sintering atmosphere on microstructure and mechanical property of sintered powder metallurgy 316L stainless steel. Mater. Des..

[50] Lozada L., Castro F. Controlled densification of boron-containing stainless steels. Proceedings of the Advances in Powder Metallurgy & Particulate Materials.

